# System approaches to study root hairs as a single cell plant model: current status and future perspectives

**DOI:** 10.3389/fpls.2015.00363

**Published:** 2015-05-19

**Authors:** Md Shakhawat Hossain, Trupti Joshi, Gary Stacey

**Affiliations:** ^1^Division of Plant Sciences and Biochemistry, National Center for Soybean Biotechnology, Christopher S. Bond Life Sciences Center, University of Missouri, Columbia, MO, USA; ^2^Department of Computer Science, Informatics Institute and Christopher S. Bond Life Sciences Center, University of Missouri, Columbia, MO, USA

**Keywords:** root hair, single cell, rhizobium, soybean, systems biology

## Abstract

Our current understanding of plant functional genomics derives primarily from measurements of gene, protein and/or metabolite levels averaged over the whole plant or multicellular tissues. These approaches risk diluting the response of specific cells that might respond strongly to the treatment but whose signal is diluted by the larger proportion of non-responding cells. For example, if a gene is expressed at a low level, does this mean that it is indeed lowly expressed or is it highly expressed, but only in a few cells? In order to avoid these issues, we adopted the soybean root hair cell, derived from a single, differentiated root epidermal cell, as a single-cell model for functional genomics. Root hair cells are intrinsically interesting since they are major conduits for root water and nutrient uptake and are also the preferred site of infection by nitrogen-fixing rhizobium bacteria. Although a variety of other approaches have been used to study single plant cells or single cell types, the root hair system is perhaps unique in allowing application of the full repertoire of functional genomic and biochemical approaches. In this mini review, we summarize our published work and place this within the broader context of root biology, with a significant focus on understanding the initial events in the soybean-rhizobium interaction.

## Why Root Hairs are an Excellent, Single-cell, Plant Model for Systems Biology?

A root hair is a single cell (Wan et al., 2005; Brechenmacher et al., 2009, 2012; Libault et al., 2010a; Qiao and Libault, 2013), structurally simple and tubular outgrowth of root epidermal cells (Grierson et al., 2014). Root hairs have a huge absorptive surface area (Hofer, 1991), evolved in order to allow the plant to take up water, nutrients and minerals (Minorsky, 2002). They are also a major route for plant-microbe interactions (Oldroyd, 2001). For example, legume-rhizobium interactions that lead to the formation of a new organ, the nodule, where biological N_2_-fixation takes place (Oldroyd and Dixon, 2014).

In order to adapt to environmental changes and respond to morphological or developmental stimuli, plant cells have evolved complex regulatory networks integrating the response at a variety of levels; such as DNA, RNA, proteins, metabolites and small molecules (Pu and Brady, 2010). While some plant genomes can encode over 50,000 genes, it is clear that many genes are specifically expressed in only a few organs, tissues or cell types. However, it can be technically very challenging to measure the levels of genes, proteins or metabolites in a specific cell type (Rogers et al., 2012). Hence, most studies measure these components in whole plants or tissues, resulting in an averaging of the responses occurring within all cells. In order to overcome these sampling issues, the soybean root hair was proposed as a single cell plant model (Libault et al., 2010a). The size and thickness of the soybean root allows root hair cells to be isolated easily after freezing in liquid nitrogen. The result of this procedure is pure root hair preparations in gram quantities allowing the full repertoire of functional genomic methods to be applied (Wan et al., 2005; Brechenmacher et al., 2009, 2012; Libault et al., 2010a; Nguyen et al., 2012; Qiao and Libault, 2013).

**FIGURE 1:**
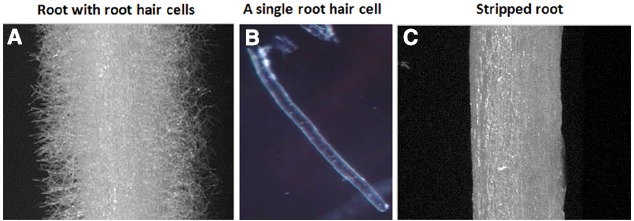
**Soybean root hairs and stripped roots. (A)** Root with root hairs, **(B)** A representative of single cell root hair and **(C)** stripped roots (i.e., root hairs removed from roots).

**FIGURE 2:**
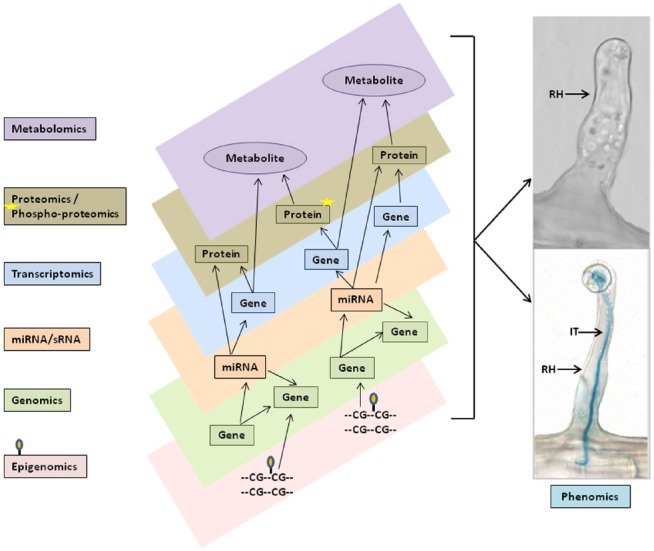
**Schematic representation of multi- omics data integration and networks for single cell phenomic studies.** Varieties of multi-omics data set, such as genomics and epigenomics (i.e., DNA methylation), miRNAs/sRNAs, transcriptomics, proteomics and phosphoproteomics, and metabolomics (left flow chart) were generated from soybean single cell root hairs and predicted to be combined in the *in silico* computational models (different layers in center panel) to perform multi-omics data integration, networks and pathway analysis for biological model hypothesis to phenomics studies (right panel). RH, Root hair; IT, Infection thread.

Studies of plant root hairs are not new. A literature search identified more than 1,300 articles covering various aspects of root hair biology, including studies of root hair elongation, tip growth, polarized cell expansion, endomembrane trafficking, cytoskeletal organization and cell wall modifications in model and crop plants; such as, *Arabidopsis*, *Lotus japonicus*, rice, corn, barley, and tomato (http://www.iroothair.org/; Foreman and Dolan, 2001; Karas et al., 2005; Yokota et al., 2009; Hossain et al., 2012; Peña et al., 2012; Kwasniewski et al., 2013; Grierson et al., 2014). However, recent advances with our methods of single cell root hair isolation in soybean (Figure 1; Libault et al., 2010a; Qiao and Libault, 2013), along with the availability of the soybean genome sequence (Schmutz et al., 2010) and high-throughput sequencing, proteomic, metabolomic and epigenetic technologies make the soybean root hair system particular attractive for detailed, systems-level studies. The ultimate goal is to use this information for computational prediction and integration of big data sets for network analysis of plant cell function (Figure 2; Foreman and Dolan, 2001; Wan et al., 2005; Brechenmacher et al., 2009, 2012; Kwasniewski et al., 2010; Libault et al., 2010a; Peña et al., 2012; Grierson et al., 2014).

## What have We Learned about Root Hairs Using System Approaches?

The study of a single cell system provides significantly higher resolution and sensitivity when various functional genomic methods are applied. This has been amply demonstrated by studies in *Arabidopsis* that characterized the transcriptome, proteome and metabolome of various root cell types (Pu and Brady, 2010; Rogers et al., 2012; Moussaieff et al., 2013; Misra et al., 2014). In our laboratory, over the past few years, the full repertoire of functional genomic methods has been applied to studies of soybean root hairs as a single cell plant model (Wan et al., 2005; Brechenmacher et al., 2009; Libault et al., 2010b; Qiao and Libault, 2013). From these efforts, one could argue that, the soybean root hair cell is one of the best characterized cell types in plant biology.

## Root Hair Transcriptome

A number of genome-wide transcriptome profiling studies have been published using root hairs from several model and crop plants (Jones et al., 2006; Kwasniewski et al., 2010; Libault et al., 2010b; Lan et al., 2013; Libault, 2013; Breakspear et al., 2014). For example, in *Arabidopsis* and barley, researchers used this cell type to study transcriptional regulation mostly focused on root hair morphogenesis, cell fate, cellular growth and differentiation. Since root hairs expand by polar growth, along with pollen tubes, they serve as a model to study this distinctive growth process (Campanoni and Blatt, 2007).

However, in legumes, root hairs are also the primary site for rhizobial infection and, therefore, several studies have sought to define the early events in this infection process. Libault et al. (2010b) studied soybean root hair infection by the symbiotic bacterium *Bradyrhizobium japonicum*. This study used two combined platforms (Affymetrix and Illumina sequencing) and identified 1,973 genes that were differentially expressed in response to bacterial infection, including those involved in the initial rhizobial symbiotic signal, lipo-chitooligosaccharide (Nod) factor perception, plant defense response, modification of cell wall composition, signal transduction, basic metabolic processes, and hormonal regulation (Libault et al., 2010b). Very similar findings came from a more recent investigation in the model legume, *Medicago truncatula* (Breakspear et al., 2014). A microarray based transcriptomics approach identified hundreds of genes regulated in root hair cells in response to *Sinorhizobium meliloti* and bacterial Nod factor application. A comparison of these two studies revealed ∼370 genes differentially regulated by rhizobial inoculation in the two legume species. Among genes responding in both species were those shown previously to be critical for the legume-rhizobial interaction; including, *NIN* (Nodule Inception), *PUB1* (Plant U-box Protein 1), *VPY* (Vapyrin), *RPG* (Rhizobium-directed Polar Growth), *NSP1* (Nodulation Signaling Pathway 1), *NSP2* (Nodulation signaling Pathway 2), *NPL1* (Nodulation Pectate Lyase 1), *FLOT4* (Flotillin-like protein 4), *ERN1* (Ethylene Response Factor Required for Nodulation1), *ERN2* (Ethylene Response Factor Required for Nodulation2), *NFYA1* (Nuclear transcription factor Y subunit A-1), and *NMN1* (Nucleolar/Mitochondrial protein involved in Nodulation) (Breakspear et al., 2014). These findings emphasize the utility of root hair studies to identify key genes involved in the rhizobial symbiosis.

## Root Hair Proteome

While transcriptome studies are rather common, fewer studies have focused on the root hair proteome (Wan et al., 2005; Brechenmacher et al., 2009, 2012; Pang et al., 2010; Nestler et al., 2011; Subramanian and Smith, 2013). One can argue that, while mRNA profiling provides a picture of the potential functions in the cell, only the proteome can give you a true picture of which of these functions are likely occurring. The first report on the soybean root hair proteome focused on providing a protein reference map of this single cell type (Brechenmacher et al., 2009) using two-dimensional-polyacrylamide gel electrophoresis (2D-PAGE), augmented by multidimensional protein identification technology (MudPIT). This study identified 1,492 proteins involved in basic cell metabolism, water and nutrient uptake, vesicle trafficking, and hormone and secondary metabolism. A later study, using the Accurate Mass and Time (AMT) tag approach combined with liquid chromatography-tandem mass spectrometry (LC-MS/MS), identified a total of 5,702 proteins from soybean root hair cell preparations (Brechenmacher et al., 2012). Both studies reported similar functional categories of proteins. A recent study focused on root hairs isolated from the monocot maize with proteins separated by 1-dimensional PAGE and then subjected to nano LC-MS/MS (Nestler et al., 2011). This study identified 2,573 abundant proteins in maize root hair cells. Interestingly, a comparison of the soybean (dicot) and maize (monocot) datasets identified 252 conserved proteins pointing to functionally conserved, root hair functions in these disparate species.

In order to specifically address protein changes occurring in root hairs upon rhizobial inoculation, Wan et al. (2005) utilized 2-D PAGE to separate proteins, which were then identified by matrix-assisted laser desorption/ionization-time of flight (MALDI-TOF) MS. This studied identified 37 proteins including enzymes, such as chitinase and phosphoenolpyruvate carboxylase, that appeared to be specific to root hairs (relative to roots stripped of the root hairs), as well as peroxidase, phenylalanine-ammonia lyase, lectin, phospholipase D and phosphoglucomutase, whose expression changed significantly upon rhizobial inoculation. It was previously shown that these proteins played significant roles in root hair deformation, infection and legume nodulation in response to bacterial infection (Halverson and Stacey, 1985; Díaz et al., 1989; Estabrook and Sengupta-Gopalan, 1991; Cook et al., 1995; van Rhijn et al., 1998; den Hartog et al., 2001; Lepek et al., 2002; Mitra and Long, 2004; Yan et al., 2015b).

## Root Hair Phosphoproteome

It is now clear that many of the initial events in Nod factor perception and the plant response to rhizobial infection involves activation of a variety of protein kinases (Antolín-Llovera et al., 2014; Liang et al., 2014). Therefore, it is of interest to identify those proteins that are rapidly phosphorylated after rhizobial inoculation. Modern MS-based methods for phosphoproteomic analysis allow for such global analyses. For example, Rose et al. (2012) analyzed the phosphoproteome of *M. truncatula* roots after inoculation with *S. meliloti*. However, again, although this report identified a variety of interesting proteins, the results represent an average of the whole root response, not that of specific cells. Hence, our laboratory undertook a similar study to specifically examine the phosphoproteome of soybean root hair cells subsequent to rhizobial inoculation (Nguyen et al., 2012). Again, this is an ideal cell type since it is the site of the initial interaction and penetration of the plant root by the rhizobial symbiont. The root hair phosphoproteome was compared to that of roots stripped of their root hairs. In order to provide accurate quantification of peptide levels, each was labeled with an isobaric tag (eight plex) using the iTRAQ (isobaric tags for relative and absolute quantification) method, followed by phosphopeptide enrichment and LC-MS/MS analysis. A total of 1,625 unique phosphopeptides, spanning 1,659 non-redundant phosphorylation sites, were detected from 1,126 soybean phosphoproteins from both root hairs and stripped roots. Among the identified phosphopeptides, the levels of 273, belonging to 240 phosphoproteins, were found to be significantly regulated upon *B. japonicum* infection suggesting a complex network of kinase substrate and phosphatase-substrate interactions in response to rhizobial inoculation (Nguyen et al., 2012). Proteins predicted to play a role in signal transduction (e.g., protein kinases, protein phosphatases, protein phosphatase inhibitors, and G protein-related proteins) and those involved in hormone signaling were among those whose phosphorylation was specifically affected by *B. japonicum* inoculation. The identified phosphoproteins and phosphorylation site data were deposited and are available at the Plant Protein Phosphorylation Database (P^3^DB; http://digbio.missouri.edu/p3db/).

## Root Hair Metabolome

Similar to transcriptomics and proteomics, responses and measurement of metabolites from multi-cellular tissues, organs or the whole plant could give misleading information if specific metabolism is confined to only a few cells or a single cell type. Until recently, a single analytical platform was not available that could measure a significant number of metabolites from a single cell (Fukushima et al., 2009). However, extant methods could be applied to soybean root hairs given our ability to isolate this cell type in a pure form and in quantity (Libault et al., 2010a). Again, the legume-rhizobium interaction was an obvious target for such analyses given the relevance of the root hair system and a variety of publications implicating specific metabolites as playing important roles. A variety of secondary products (e.g., flavonoids), as well as various hormones, were shown to be important regulators of the nodulation process (Matamoros et al., 2006; Gibson et al., 2008; Ding and Oldroyd, 2009). For example, formation of the infection thread by which rhizobia gain entry into the plant root hair cell might require a significant change in the metabolism of both the symbiont and host.

Brechenmacher et al. (2010) investigated the root hair metabolome in an effort to identify metabolites whose levels changed significantly during the first 48 h after rhizobial inoculation. Metabolites were analyzed using both gas chromatography-mass spectrometry (GC-MS) and ultra-performance liquid chromatography-quadrupole time of flight-mass spectrometry (UPLC-MS). Using these combined approaches, a total of 2,610 metabolites were identified in soybean root hair cells. Among these metabolites, 166 were found to be significantly regulated in response to rhizobial infection, including various (iso)flavonoids, amino acids, fatty acids, carboxylic acids, and various carbohydrates, indicating major metabolic changes occurring during *B. japonicum*-root hair interactions. Among these metabolites was trehalose, an α-linked disaccharide of glucose, which has been implicated in a variety of plant processes, including resistance to osmotic stress (Iturriaga et al., 2009). Trehalose levels increased significantly in root hairs upon inoculation by *B. japonicum*. However, through the use of various mutants of *B. japonicum* blocked in the synthesis of trehalose, the majority of this disaccharide could be attributed to the bacteria and did not appear to be predominantly derived from the plant host. The authors postulated that trehalose synthesis by *B. japonicum* may be important to allow the bacteria to survive what may be a stressful osmotic environment within the plant.

## Root Hair Small RNAome

MicroRNAs (miRNAs) are small non-protein coding endogenous RNAs, typically 21-24 nucleotides in length. In the past decade, miRNAs have been shown to be key players controlling gene expression by transcript cleavage or translational inhibition in a wide variety of plant biological processes, including growth and development, disease, stress and plant microbe interactions (Kidner and Martienssen, 2005; Xiao and Rajewsky, 2009; Lauressergues et al., 2012; Sunkar et al., 2012; Weiberg et al., 2013). Indeed, several studies documented an important role for miRNAs in regulated gene expression during the legume nodulation process (Combier et al., 2006; Subramanian et al., 2008; Lelandais-Brière et al., 2009; Wang et al., 2009; Joshi et al., 2010; Li et al., 2010; De Luis et al., 2012; Turner et al., 2013; Yan et al., 2013, 2015a). However, all of these reports came from small RNA profiling of roots and/or nodules and not from more specific studies of the initial infection process within root hairs. In order to target these very early stages of rhizobial-host interaction, Yan et al. (2015b) utilized *B. japonicum* infected soybean root hair single cells and roots stripped of their root hairs to generate both small RNA and degradome libraries. Sequencing of three small RNA libraries from inoculated root hairs, stripped roots and mock inoculated control samples identified a total of 114 miRNAs. Among these, 22 were found to be novel miRNAs. Comparative analysis of miRNA abundance identified 66 miRNAs that were differentially expressed between root hair and stripped roots. A total of 48 miRNAs were differentially regulated in root hairs in response to bacterial infection when compared to the un-infected control root hairs. Sequencing of a Parallel Analysis of RNA Ends (PARE; i.e., degradome) library from similar tissues revealed a total of 405 soybean mRNA targets. This method identifies new 5′-mRNA ends presumably arising from miRNA-mediated cleavage (German et al., 2009; Zhai et al., 2014). The mRNA targets identified were predicted to encode transcription factors or proteins involved in protein modification, protein degradation and enzymes in various hormonal pathways. The root hair data set represents an important starting point for in depth analysis of the role that specific miRNAs may be playing in the legume-rhizobial symbiosis.

## Bioinformatics and Data Integration for Network Analysis and Modeling

Next generation sequencing technologies have empowered researchers to conduct experiments on a whole genome scale and have the potential to completely revolutionize biological research. Big Data have been generated in all domains including transcriptomics (RNA-seq), proteomics, metabolomics, epigenomics, microRNA/smallRNA, and genomic variations, including single nucleotide polymorphisms (SNP) and insertion/deletions (InDels; Mochida and Shinozaki, 2010; Brauer et al., 2014). Most lab experiments now generate anywhere from a few GB to several TB of raw and analyzed data, and have a need to overlay different types of data and information to get a comprehensive understanding. All of these data provide valuable insights into a systems-level understanding of the biology of an organism and need to be mined in an innovative and integrative manner. This is posing a new challenge for researchers and mandating the development of comprehensive, efficient informatic platforms and web resources to facilitate data sharing and collaboration among the research community, while providing advanced techniques for multi-omics integration, computational analysis, and hypothesis generation.

Most databases and available tools can handle analysis of a single -omics data-type, but analysis of multiple omics data-types presents a major challenge. No tools are currently available that provide a systematic solution to this problem. In most cases, only by integrated analysis of the expression of mRNA, proteins, metabolites along with miRNA/sRNA, etc (Figure 2) can we draw conclusions about all the involved regulatory mechanisms. With multi-omics data integration techniques, we can gain better insight into the network modules derived and, supported by experimental evidence, utilize this information to build *in silico* computational models for understanding the underlying mechanisms and to generate new hypotheses. The *in silico* models provide templates that automate the identification and generation of network modules by incorporating differentially expressed genes, proteins, and metabolites into the function and pathway enrichment analysis. The models can be visualized using multiple layers of platforms such that data from transcriptomics, proteomics, and metabolomics can each be visualized simultaneously on separate platform layers as shown in Figure 2. This presentation enables rapid comparisons that can be further enriched by incorporating available experimental data, literature references, and user inputs to cross-validate results. Users can start with their own hypothesis and add the multi-omics evidence stored in the associated database to continue building the modules. Such *in silico* modeling systems can provide researchers with additional avenues for generating and testing a specific hypothesis by incorporating diverse varieties of data. Bioinformatics plays a key and essential role in bringing these pieces together in the form of one-stop-shop web resources, such as the soybean knowledge base (SoyKB; Joshi et al., 2014, 2012), and facilitating the ability of researchers to get a more complete understanding of the underlying molecular mechanisms.

## Conclusion

Our planet is facing increasing challenges related to environment, food security, non-renewable energy sources, water availability and overall sustainability. Plant biological research and its application in agriculture have the potential to mitigate many of these challenges. Thus, we need to have a better understanding of plant biology at a systems level, ultimately reaching the point where computational models can predict biological outcomes. Irrespective of the popularity of system biology, we are still a long way from achieving this level of understanding. Among the obstacles is the functional complexity of cellular/organismal regulatory networks, as well as our ability to deal with Big Data, especially integration of disparate data types. There is a need to simplify our systems to allow for clearer conclusions, while increasing the resolution and sensitivity of our analysis, without losing contact with real world relevance. The root hair single cell system provides one such route for the study of plant processes. The use of the diverse “omics” data sets developed from the soybean root hair as a single cell type provides an opportunity for comprehensive network analysis and ultimately will help to build an integrated predictive model. Besides the inherent interest of the root hair cell, the hope is that as an illustrative model system, the soybean root hair cell system can contribute to an overall greater understanding of plant biology leading ultimately to improvements in agriculture.

### Conflict of Interest Statement

The authors declare that the research was conducted in the absence of any commercial or financial relationships that could be construed as a potential conflict of interest.
